# Investigating the association between recorded smoking cessation interventions and smoking cessation in people living with cardiovascular disease using UK general practice data

**DOI:** 10.1186/s12875-025-02843-9

**Published:** 2025-05-01

**Authors:** Angela Difeng Wu, Nicola Lindson, Rafael Perera, Min Gao, Paul Aveyard, Rachna Begh, Jamie Hartmann-Boyce

**Affiliations:** 1 Nuffield Department of Primary Care Health Sciences, University of Oxford, Oxford, UK; 2 Department of Health Promotion and Policy, University of Massachusetts, Amherst, MA USA

## Abstract

**Background:**

Smoking significantly increases the risk of cardiovascular diseases (CVD), yet quitting smoking after diagnosis of CVD can mitigate further negative impacts. However, encouraging smoking cessation remains a challenge for General Practitioners (GPs) with concerns regarding mental health. Since 2004, the UK’s Quality and Outcomes Framework (QOF) incentivises GP smoking cessation support. Despite this, a significant proportion of individuals diagnosed with CVD continue to smoke after diagnosis. This study aims to investigate the frequencies and types of smoking cessation interventions offered to people with CVD (defined as coronary heart disease (CHD) and stroke), with and without mental illness, and assess their association with successful cessation.

**Methods:**

This retrospective cohort study examined adults diagnosed with CHD or stroke using the QResearch general practice records database (1996–2019). We evaluated the frequency and types of smoking cessation interventions documented in patients’ records, including education, brief interventions, pharmacological support, referrals, and counselling. Logistic regression assessed the relationship between recorded interventions and smoking abstinence rates within the one-year post-index event, considering QOF incentives and mental illness presence.

**Results:**

While smoking cessation education was common in general practice settings, prescriptions for nicotine replacement therapy or other evidence-based interventions were comparatively low. CHD and stroke populations showed a significant association between any intervention and smoking cessation within one year (CHD: OR 1.41, 95% CI 1.36–1.45; stroke: OR 1.49, 95% CI 1.43–1.55). Education consistently correlated with higher cessation likelihoods, while other interventions were linked to lower rates. Individuals with common and serious mental illness were less likely to quit, irrespective of intervention. QOF implementation led to increased documentation of advice but not intensive support or treatment, with pre-QOF interventions associated with significantly increased abstinence likelihoods (CHD: OR 5.09, 95% CI 4.84–5.35; stroke: OR 4.44, 95% CI 4.07–4.86).

**Conclusions:**

Financial incentives for GP smoking cessation support outlined in QOF may not suffice to enhance methods that are more efficacious or improve cessation rates, especially among people with mental illness. Practical strategies that provide tangible support and treatment are needed for CVD patients, including those with mental illness, to facilitate successful cessation.

**Supplementary Information:**

The online version contains supplementary material available at 10.1186/s12875-025-02843-9.

## Background

Smoking remains one of the leading behavioural causes of CVD and CVD mortality [1]. People who currently smoke or have recently quit face a substantially higher risk of CVD compared to people who have never smoked or people who used to smoke [2]. Smoking cessation has been identified as a crucial intervention for secondary prevention of CVD, as evidenced by studies demonstrating a decrease in the risk of secondary CVD events following smoking cessation [3]. A recent Cochrane systematic review found that people who stopped smoking after their CVD diagnosis were a third less likely to die from heart disease or stroke and a third less likely to have another heart attack or stroke [3].

While some people who quit do so without support [4], quit attempts are more likely to be successful when supported by evidence-based behavioural [5] and pharmacological interventions [6, 7, 8]. A recent component network meta-analysis looking at data from randomised controlled trials (RCTs), which examined various aspects of pharmacotherapies and e-cigarettes for smoking cessation, concluded that the most effective interventions were nicotine e-cigarettes and the smoking cessation medicines varenicline and cytisine, closely followed by combination nicotine replacement therapy (NRT), which refers to use of nicotine patch concurrently with a short-acting form of NRT (e.g. gum or lozenges) [9].

In the United Kingdom, smoking in the general population has decreased from approximately 46% in the 1970s to 12.9% in 2022 [10–14]. While a decrease in smoking rates has also been seen among adults with a long-term mental health condition– falling from 35.3% in 2013 to 2014 to 26.8% in 2018 to 2019–the smoking rate among adults with mental health conditions is more than double the rate in the general population, despite the same levels of motivation to quit [15, 16]. Additionally, mental illness is linked to an increased susceptibility to various physical health issues, including CVD [17, 18, 19]. Existing literature explores the associations between smoking cessation and mental health, smoking cessation and CVD risk, and mental health and CVD risk [20, 21, 22] separately. However, there is a gap in the literature examining all three factors simultaneously.

Even though most people who smoke report that they want to quit [23], many continue smoking because they consider smoking a coping mechanism for stress, offering them mental health benefits [24, 25]. This misperception can deter individuals from quitting, as they fear a decline in mental health. Moreover, health professionals may hesitate to recommend smoking cessation to certain patients, concerned about the perceived impact on mental well-being [26, 27]. Recognising that physicians may hesitate to promote smoking cessation for people with mental health concerns, it becomes essential to account for the influence of mental health when advocating cessation for those diagnosed with both CVD and mental illnesses.

In the UK, the initiation of the Quality and Outcomes Framework (QOF) in April 2004 marked the first instance of a financial incentive for GPs to document whether people who smoked had been offered cessation advice in their medical records, with an approximate value of £4,500 per general practice per year, based on the size of the practice [28]. This incentivisation appeared effective in increasing the documentation of smoking cessation advice and recorded referrals to smoking cessation services by GPs [29]. However, this surge in advice provision did not translate into a concurrent increase in the prescription of NRT or stop smoking medications such as bupropion and varenicline; neither of which were incentivised by QOF [29].

Thus, while the introduction of QOF heightened the likelihood of documented smoking cessation advice, it may not have increased the actual rate of advice provision. There is clear evidence to show that support in how to quit is more motivating and successful than advice to quit [30]. The meta-analysis concluded that directing patients to available cessation support options significantly increased the likelihood of engaging in smoking cessation interventions compared to only informing them about the harms of smoking and the benefits of stopping [30]. However, under the current model, the advice documentation code is much more commonly used than offering support or treatment in the general population [29]. A study compared patient recall of receiving smoking cessation advice in a nationwide survey on British National Health Service (NHS) experiences with rates reported in electronic primary care medical records [31]. The findings indicated that patient smoking cessation advice recall and electronic records closely aligned in 2004. However, while records of interventions in electronic records surged in 2005 and 2008, there was no corresponding increase in patient recall. Similarly, a study focusing on people with mental illness noted that increases in recorded smoking cessation advice rates in primary healthcare records from 2007 to 2014 were not accompanied by increases in the prescription of smoking cessation medications, recorded quit attempts, or changes in smoking status [32].

In light of this, this study aimed to investigate the frequencies and types of smoking cessation interventions offered to people with CHD or stroke, with and without mental illness, using a large UK-based primary care record database. Specifically, the study aimed to assess, after the introduction of QOF, whether records of these interventions were associated with higher success rates for smoking cessation.

## Method

### Study design

A retrospective cohort design, including adults (> 18 years of age) diagnosed with CHD or stroke within the QResearch database from January 1st 1996 to December 31st 2019. We did not use data collected after 2019 due to any potential impact of the coronavirus pandemic (COVID-19). This is not a clinical trial, and therefore does not have a trial registration number, however the protocol was made publicly available prior to data analysis [33] and the analysis code is hosted online [34].

### Data source

We used anonymised UK primary care patient records from the QResearch primary care research registry (http://www.qresearch.org). QResearch is one of the largest general practice databases in the UK, containing over 35 million people registered with 1805 general practices [35].

### Participants

We used data from adults (> 18 years) who were smoking when diagnosed with either coronary heart disease (CHD) or stroke, and categorised them based on whether they had accompanying psychiatric disorders. For this analysis, we defined CVD as encompassing CHD or stroke. We defined CHD using Systematized Nomenclature of Medicine (SNOMED) codes, encompassing people with confirmed myocardial infarction or identifiable evidence from electrocardiograms and imaging, along with corresponding International Classification of Diseases (ICD) coding. Stroke cases were identified through SNOMED codes indicating ischemic, haemorrhagic, or unspecified stroke, supported by relevant ICD coding. To be eligible for inclusion, participants had to be registered with their GP practice for at least one year before their diagnosis and have at least one year of follow-up data within the QResearch database. We classified people as smoking at the time of diagnosis if their smoking status was documented within the five years prior to their CHD or stroke diagnosis. For the assessment of mental health conditions, people were categorised into common mental illness (CMI) if they had recorded SNOMED codes indicating conditions such as depression, generalized anxiety disorder, panic disorder, obsessive-compulsive disorder, body dysmorphic disorder, post-traumatic stress disorder, or social anxiety disorder within the year before or after their CVD event. For serious mental illness (SMI), including affective and non-affective psychosis, people were classified as having SMI if they had ever been documented with the corresponding SNOMED code. If people lacked SNOMED codes for either SMI or CMI, we presumed they were without mental illness.

### Variables

#### Exposure

We defined and classified intervention types using SNOMED codes, with a detailed listing provided in the Appendix. An intervention was deemed present if there was a documented attempt to intervene, regardless of whether the patient accepted it. For example, the code “intervention referral offer” was categorised as ‘yes’ regardless of the patient’s acceptance of the referral. We examined whether each intervention was offered within the initial year following the index event of stroke or CHD. Interventions were sorted into five categories: referral offers to stop smoking services, brief interventions, smoking education, offers of pharmacological intervention, and smoking cessation counselling. We broadly defined the meaning behind each intervention type as seen in Table 1. While it is not possible to confirm if the GPs who recorded the codes inferred the same meaning as us, this follows the current literature on smoking interventions.

**Table 1:** Common smoking cessation intervention descriptions

Intervention Category	Description
Referral offer to stop smoking services	Involves referring people who smoke to specialised services that provide support and resources to help them quit smoking.
Brief intervention	A short, evidence-based, structured conversation about health behaviours. It can take from 30 s to a couple of minutes and is mainly about giving people information or directing them where to go for further help.
Smoking education	Providing information about the risks of smoking and the benefits of quitting.
Offer of pharmacological intervention	The use of medications to help people who smoke quit. There are several types of medications available, including NRT, varenicline, and bupropion.
Smoking cessation counselling	Providing support and strategies to help people who smoke quit.

#### Outcome

To construct the smoking cessation variable, the date that abstinence was first recorded was utilised, meeting the following specific criteria:

- The patient had a record of ex-smoker or non-smoker status.
- The subsequent two smoking records were also coded as ex-smoker or non-smoker (unless data were missing).
- There were no subsequent positive records of smoking.

We adopted a conservative approach where we assumed people were still smoking unless there was a record indicating otherwise.

### Covariates

Covariates included age, sex, socio-economic status, ethnicity, timing of the index event relative to the introduction of QOF, hypertension, diabetes status, atrial fibrillation, lung cancer, head and neck cancer, heart failure, chronic obstructive pulmonary disease (COPD), asthma, terminal illness, and medications for secondary prevention of CHD or stroke (antiplatelet agents, statins, beta-blockers, ACE inhibitors).

### Statistical analysis

We conducted all analyses in Stata 16. We tracked smoking cessation interventions annually for up to five years post-diagnosis of CVD. Data included intervention frequency, timing of first intervention, smoking abstinence achievement, population-level rates of unaided cessation and new abstinence per year. We identified the most common intervention across the five-year follow-up period.

We used logistic regression to analyse the effect of smoking cessation interventions on quitting within one year of the index event. All covariates were included in the model, and we amalgamated all intervention types into one model to measure independent impact of each intervention on smoking cessation.

### Subgroup and sensitivity analysis

We conducted subgroup analyses stratifying intervention types and frequencies by the presence of common or serious mental illness. We also conducted a sensitivity analysis comparing the impact of recorded intervention on smoking cessation pre versus post QOF.

### Missing data

We planned a complete case analysis for the covariates sex, age, socioeconomic status, hypertension, and lipids, unless at least 5% of the population had missing data. For the covariates diabetes status, diagnosis of CMI, SMI, head and neck cancer, asthma, COPD, terminal illness and medications for secondary prevention of CHD or stroke, we assumed missing data meant the covariate was not present for the individual.

### Ethics approval and funding

The project has been independently peer reviewed and received ethics approval from the QResearch Scientific Board (reference OX57 under REC 18/EM/0400 from the Trent Multi-Centre Research Ethics Committee. The protocol was published on Open Science Framework at https://osf.io/7k3zj/. This work was funded by the British Heart Foundation Studentship Grant FS/19/78/34716.

### Differences from protocol

We made the post-hoc decision to refine the population for our primary analysis looking at the impact of smoking cessation interventions on quitting smoking. We restricted it to people diagnosed after 2004, coinciding with the introduction of QOF. We made this decision so that the data would reflect the current state of GP behaviours, aligning with our related aim and rationale.

## Results

### Trends in smoking cessation interventions

#### Population with coronary heart disease

A total of 158,470 people diagnosed with CHD were documented as smoking prior to their first heart attack (index event) (Table 2). Among this cohort, 64% experienced their index event post-implementation of the Quality and Outcomes Framework (QOF). Within the first year following their index event, 89,707 people (56.6%) received at least one smoking cessation intervention. Of those without recorded interventions, 22% achieved smoking cessation within one year of diagnosis, whereas 39,199 people (45.9%) who received interventions successfully quit within the same timeframe.

**Table 2:** Baseline characteristics of people with heart disease spilt one-year intervention recorded

Characteristic	Whole Cohort (*n* = 158,470)	Intervention Recorded (*n* = 89,707)	No Intervention Recorded (*n* = 68,763)
**Age**			
Aged 18/30	112 (0.1%)	18 (< 1%)	94 (0.1%)
Aged 31/40	3521 (2.2%)	1671 (2.0%)	1850 (2.5%)
Aged 41/50	22,513 (14.2%)	12,004 (14.0%)	10,509 (14.4%)
Aged 51/60	44,374 (28.0%)	23,392 (27.4%)	20,982 (28.7%)
Aged 61/70	46,629 (29.4%)	24,566 (28.7%)	22,063 (30.2%)
Aged 71/80	30,839 (19.5%)	17,014 (19.9%)	13,825 (18.9%)
Aged 81/90	9691 (6.1%)	6232 (7.3%)	3459 (4.7%)
Aged 91/110	791 (0.5%)	551 (0.6%)	240 (0.3%)
**Female**	53,743 (33.9%)	28,965 (33.9%)	24,778 (33.9%)
**Ethnicity**			
White	104,033 (65.6%)	61,880 (72.4%)	42,153 (57.7%)
Indian	1999 (1.3%)	1273 (1.5%)	726 (1.0%)
Pakistani	2213 (1.4%)	1367 (1.6%)	846 (1.2%)
Bangladeshi	1626 (1.0%)	959 (1.1%)	667 (0.9%)
Other Asian	1055 (0.7%)	661 (0.8%)	394 (0.5%)
Caribbean	1137 (0.7%)	730 (0.9%)	407 (0.6%)
Black African	518 (0.3%)	359 (0.4%)	159 (0.2%)
Chinese	153 (0.1%)	96 (0.1%)	57 (0.1%)
Other^a^	1595 (1.0%)	1004 (1.2%)	591 (0.8%)
Missing	44,142 (27.9%)	17,119 (20.0%)	27,023 (37.0%)
**Abstinence within one year of index event**	55,441 (35.0%)	39,199 (45.9%)	16,242 (22.2%)
**Prescription for CVD medication** ^b^	156,135 (98.5%)	84,515 (98.9%)	71,620 (98.1%)
**Mental Illness**			
**No Mental Illness**	132,039 (83.3%)	72,520 (80.8%)	59,519 (86.6%)
**Common Mental Illness** ^c^	23,805 (15%)	15,561 (17.3%)	8244 (11.9%)
**Serious Mental Illness** ^d^	2626 (1.7%)	1626 (1.9%)	1000 (1.5%)
**Lung Cancer**	5836 (3.7%)	2578 (3.0%)	3258 (4.5%)
**Head and Neck Cancer**	586 (0.4%)	281 (0.3%)	305 (0.4%)
**COPD**	36,971 (23.3%)	19,591 (22.9%)	17,380 (23.8%)
**Asthma**	23,414 (14.8%)	13,110 (15.3%)	10,304 (14.1%)
**Heart Failure**	29,325 (18.5%)	14,663 (17.2%)	14,662 (20.1%)
**Hypertension**	24,323 (15.3%)	45,702 (53.5%)	38,914 (53.3%)
**Terminal Illness**	548 (0.3%)	223 (0.3%)	325 (0.4%)
**Atrial Fibrillation**	24,323 (15.3%)	12,646 (14.8%)	11,677 (16.0%)
**Index Event after Introduction of QOF in 2004** ^e^	101,259 (63.9%)	73,707 (86.3%)	27,552 (37.7%)
**BMI Category**			
Underweight	5341 (3.4%)	2585 (3.0%)	2756 (3.8%)
Normal weight	39,686 (25.0%)	20,981 (24.6%)	18,705 (25.6%)
Overweight	48,309 (30.5%)	26,715 (31.3%)	21,594 (29.6%)
Obese	65,134 (41.1%)	35,167 (41.2%)	29,967 (41.0%)
**Type 1 Diabetes**	3184 (2.0%)	1517 (1.8%)	1667 (2.3%)
**Type 2 Diabetes**	46,261 (29.2%)	25,424 (29.8%)	20,837 (28.5%)
**Townsend quintile**			
1 Least deprived	33,064 (20.9%)	18,225 (21.3%)	14,839 (20.3%)
2	33,264 (21.0%)	18,253 (21.4%)	15,011 (20.6%)
3	34,661 (21.9%)	18,406 (21.5%)	16,255 (22.3%)
4	32,450 (20.5%)	17,173 (20.1%)	15,277 (20.9%)
5 Most Deprived	25,031 (15.8%)	13,391 (15.7%)	11,640 (15.9%)

a) Described as people who did not fit into any of the named categories.

b) Medications grouped: antiplatelet agents, statins, beta-blockers, ACE inhibitors.

c) Common mental illness includes depression, generalised anxiety disorder and panic disorder, obsessive-compulsive disorder and body dysmorphic disorder, post-traumatic stress disorder and social anxiety disorder.

d) Serious mental illness includes psychotic and non-psychotic disorders.

e) Categorised as index event occurring after March 2004.

#### Intervention subcategories

We examined the prevalence of different smoking cessation interventions documented within the initial year of the index event (Table 3). The most frequently recorded intervention was smoking cessation education, encompassing over 50% of people (83,514 people) according to their GP records. Post-QOF implementation, smoking cessation education remained predominant, with over 71% of people receiving such education within one year of their heart attack. Prior to QOF, smoking cessation education still led as the most prevalent intervention type, but was administered to only 20% of patients.

**Table 3:** Percentage of population that received each form of intervention within one year of index event

	Education	Pharma	Referral	Brief	Counselling	No Intervention
Whole Cohort (*n* = 158,470)	83,514 (52.7%)	5420 (3.4%)	4281 (2.7%)	604 (0.4%)	245 (0.2%)	68,763 (43.4%)
Abstinence within one year (*n* = 55,441)	38,610 (69.6%)	1195 (2.2%)	966 (1.7%)	138 (0.2%)	48 (0.1%)	15,108 (27.3%)
Continued smoking within one year (*n* = 103,029)	44,904 (43.6%)	4225 (4. 1%)	3315 (3.2%)	466 (0.5%)	197 (0.2%)	53,654 (52.1%)
CVD Death (*n* = 5,089)	1968 (38.7%)	74 (1.5%)	52 (1.0%)	10 (0.2%)	2 (< 1%)	2819 (55.4%)
Mental Illness						
No Mental Illness (*n* = 132,039)	67,802 (51.3%)	4061 (3.1%)	3241 (2.5%)	448 (0.3%)	179 (0.1%)	59,519 (45.1%)
Common Mental Illness (*n* = 23,805)	14,221 (59.7%)	1222 (5.1%)	937 (3.9%)	135 (0.6%)	56 (0.2%)	8244 (34.6%)
Serious Mental Illness (*n* = 2,626)	1491 (56.8%)	137 (5.2%)	103 (3.9%)	21 (0.8%)	10 (0.4%)	1000 (38.1%)
Townsend quintile						
1 Least deprived (*n* = 33,064)	17,928 (54.2%)	774 (2.3%)	555 (1.7%)	83 (0.3%)	55 (0.2%)	14,157 (42.8%)
2 (*n* = 33,264)	17,901 (53.8%)	1008 (3.0%)	866 (2.6%)	107 (0.3%)	44 (0.1%)	14,201 (42.7%)
3 (*n* = 34,661)	17,956 (51.8%)	1185 (3.4%)	922 (2.7%)	126 (0.4%)	51 (0.1%)	15,280 (44.1%)
4 (*n* = 32,450)	16,778 (51.7%)	1180 (3.6%)	928 (2.9%)	141 (0.4%)	50 (0.2%)	14,239 (43.9%)
5 Most Deprived (*n* = 25,031)	12,951 (51.7%)	1273 (5.1%)	1010 (4.0%)	147 (0.6%)	45 (0.2%)	10,886 (43.5%)
After QOF (*n* = 101,259)	72,063 (71.2%)	4919 (4.9%)	3982 (3.9%)	604 (0.6%)	225 (0.2%)	24,678 (24.4%)
Before QOF (*n* = 57,211)	11,451 (20.0%)	501 (0.9%)	299 (0.5%)	0 (0.0%)	20 (< 1%)	44,085 (77.1%)

**Fig. 1:**
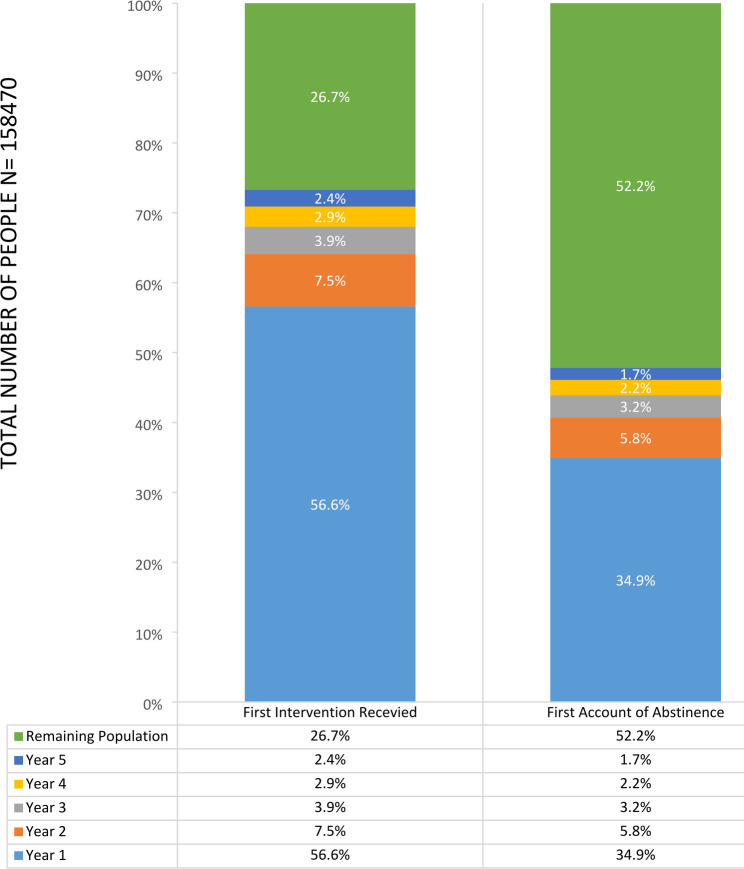
Bar chart illustrating the percentage who were first recorded as having received a smoking cessation intervention and the percentage who achieved smoking abstinence categorised by year from event of heart disease

We examined the proportion of people receiving smoking cessation interventions within the initial five years following their heart attack (Fig. 1). The rate of first intervention receipt declined annually within this population. By the fifth year, 26.7% of people had yet to receive any smoking cessation intervention since their diagnosis. Beyond the fifth year, 52.5% of the population remained smoking.

36% of people without a mental illness stopped smoking within the first year compared with 32% of people with a common mental illness, and 21% with SMI (Table 4).

**Table 4:** Baseline characteristics of people with heart disease split by presence and categories of mental illness in database

Characteristic	No Mental Illness (*n* = 132,039)	Common Mental Illness (*n* = 23,805)	Serious Mental Illness (*n* = 2626)
**Age**			
Aged 18/30	86 (0.1%)	23 (0.1%)	3 (0.1%)
Aged 31/40	2741 (2.1%)	680 (2.9%)	100 (3.8%)
Aged 41/50	17,289 (13.1%)	4971 (19.6%)	553 (21.1%)
Aged 51/60	35,655 (27.0%)	7908 (33.2%)	811 (30.9%)
Aged 61/70	40,049 (30.3%)	5881 (24.7%)	699 (26.6%)
Aged 71/80	27,072 (20.5%)	3390 (14.2%)	377 (14.4%)
Aged 81/90	8447 (6.4%)	1164 (4.9%)	80 (3%)
Aged 91/110	700 (0.5%)	88 (0.4%)	3 (0.1%)
**Female**	41,362 (31.3%)	11,343 (47.6%)	1038 (39.5%)
**Ethnicity**			
White	85,313 (64.6%)	16,949 (71.2%)	1770 (67.4%)
Indian	1793 (1.4%)	165 (0.7%)	41 (1.6%)
Pakistani	1919 (1.5%)	246 (1.0%)	48 (1.8%)
Bangladeshi	1471 (1.1%)	116 (0.5%)	39 (1.5%)
Other Asian	921 (0.7%)	114 (0.5%)	20 (0.8%)
Caribbean	977 (0.7%)	111 (0.5%)	49 (1.9%)
Black African	444 (0.3%)	46 (0.2%)	28 (1.1%)
Chinese	144 (0.1%)	6 (< 1%)	3 (0.1%)
Other^a^	1324 (1.0%)	223 (0.9%)	48 (1.8%)
Missing	37,733 (28.6%)	5829 (24.5%)	580 (22.1%)
**Abstinence within one year of index event**	47,451 (35.9%)	7429 (31.2%)	561 (21.4%)
**Current use of common secondary CVD Medication** ^b^	130,116 (98.5%)	23,458 (98.5%)	2.561 (97.5%)
**Lung Cancer**	5017 (3.8%)	742 (3.1%)	77 (2.9%)
**Head and Neck Cancer**	490 (0.4%)	84 (0.4%)	12 (0.5%)
**COPD**	29,533 (22.4%)	6719 (28.2%)	719 (27.4%)
**Asthma**	17,974 (13.6%)	4939 (20.7%)	501 (19.1%)
**Heart Failure**	24,900 (18.9%)	3981 (16.7%)	444 (16.9%)
**Hypertension**	71,354 (54.0%)	12,081 (50.7%)	1181 (45.0%)
**Terminal Illness**	459 (0.3%)	82 (0.3%)	7 (0.3%)
**Atrial Fibrillation**	21,153 (16.0%)	2910 (12.2%)	260 (9.9%)
**Index Event after Introduction of QOF** ^c^	81,460 (61.7%)	17,914 (75.3%)	1885 (71.8%)
**BMI Category**			
Underweight	4400 (3.3%)	856 (3.6%)	85 (3.2%)
Normal weight	33,909 (25.7%)	5091 (21.4%)	686 (26.1%)
Overweight	41,075 (31.1%)	6482 (27.2%)	752 (28.6%)
Obese	52,655 (39.9%)	11,376 (47.8%)	1103 (42.0%)
**Type 1 Diabetes**	2498 (1.9%)	629 (2.6%)	57 (2.2%)
**Type 2 Diabetes**	38,123 (28.9%)	7242 (30.4%)	896 (34.1%)
**Townsend quintile**			
1 Least deprived	28,887 (21.9%)	3871 (16.3%)	306 (11.7%)
2	28,399 (21.5%)	4484 (18.8%)	381 (14.5%)
3	28,700 (21.7%)	5416 (22.8%)	545 (20.8%)
4	26,176 (19.8%)	5598 (23.5%)	676 (25.7%)
5 Most Deprived	19,877 (15.1%)	4436 (18.6%)	718 (27.3%)

a) Described as people who did not fit into any of the named categories.

b) Medications grouped: antiplatelet agents, statins, beta-blockers, ACE inhibitors.

c) Categorised as index event occurring after March 2004.

#### Population with stroke

A total of 83,376 people were smoking before experiencing a stroke (their index event) (Table 5). The majority of this population (78%) encountered their index event following the implementation of QOF. Missing ethnicity data affected 22,390 people, while other baseline characteristics were adequately documented (less than 5% missing). Within the initial year of their index event, 56,169 people (67%) received a smoking cessation intervention. Among those lacking recorded interventions, 23.1% achieved smoking cessation within one-year post-stroke diagnosis.

**Table 5:** Baseline characteristics of people with CHD, spilt by whether an intervention was recorded within one year of index event

Characteristic	Whole Cohort (*n* = 83,376)	Intervention Recorded (*n* = 56,169)	No Intervention Recorded (*n* = 27,207)
**Age**			
Aged 18/30	99 (0.1%)	20 (< 1%)	79 (0.3%)
Aged 31/40	1493 (1.8%)	821 (1.5%)	672 (2.5%)
Aged 41/50	7990 (9.6%)	5252 (9.4%)	2738 (10.1%)
Aged 51/60	17,412 (20.9%)	11,584 (20.6%)	5828 (21.4%)
Aged 61/70	23,766 (28.5%)	15,752 (28.0%)	8014 (29.5%)
Aged 71/80	21,727 (26.1%)	14,724 (26.2%)	7003 (25.7%)
Aged 81/90	9871 (11.8%)	7219 (12.9%)	2652 (9.7%)
Aged 91/110	1018 (1.2%)	797 (1.4%)	221 (0.8%)
**Female**	35,874 (43.0%)	24,057 (42.8%)	11,817 (43.4%)
**Ethnicity**			
White	56,827 (68.2%)	41,222 (73.4%)	15,605 (57.4%)
Indian	715 (0.9%)	537 (1.0%)	178 (0.7%)
Pakistani	664 (0.8%)	470 (0.8%)	194 (0.7%)
Bangladeshi	467 (0.6%)	316 (0.6%)	151 (0.6%)
Other Asian	302 (0.4%)	222 (0.4%)	80 (0.3%)
Caribbean	861 (1.0%)	613 (1.1%)	248 (0.9%)
Black African	325 (0.4%)	254 (0.5%)	71 (0.3%)
Chinese	79 (0.1%)	64 (0.1%)	15 (0.1%)
Other^a^	746 (0.9%)	529 (0.9%)	217 (0.8%)
Missing	22,390 (26.9%)	11,942 (21.3%)	10,448 (38.4%)
**Abstinence within one year of index event**	30,297 (36.3%)	24,004 (42.7%)	6293 (23.1%)
**Current use of common secondary CVD Medication** ^b^	81,423 (97.7%)	55,251 (98.4%)	26,172 (96.2%)
**Mental Illness**			
**No Mental Illness**	63,959 (76.7%)	42,556 (75.8%)	21,403 (78.7%)
**Common Mental Illness** ^c^	17,525 (21%)	12,329 (21.9%)	5196 (19.1%)
**Serious Mental Illness** ^d^	1892 (2.3%)	1284 (2.3%)	608 (2.2%)
**Lung Cancer**	2947 (3.5%)	1831 (3.3%)	1116 (4.1%)
**Head and Neck Cancer**	317 (0.4%)	206 (0.4%)	111 (0.4%)
**COPD**	18,461 (22.1%)	12,663 (22.5%)	5798 (21.3%)
**Asthma**	11,206 (13.4%)	8022 (14.3%)	3184 (11.7%)
**Congestive Cardiac Failure**	9110 (10.9%)	5970 (10.6%)	3140 (11.5%)
**Hypertension**	50,378 (60.4%)	34,269 (61.0%)	16,109 (59.2%)
**Terminal Illness**	302 (0.4%)	176 (0.3%)	126 (0.5%)
**Atrial Fibrillation**	14,700 (17.6%)	10,276 (18.3%)	4424 (16.3%)
**Index Event after Introduction of QOF** ^e^	65,078 (78.1%)	51,208 (91.2%)	13,870 (51.0%)
**BMI Category**			
Underweight	3934 (4.7%)	2525 (4.5%)	1409 (5.2%)
Normal weight	23,426 (28.1%)	16,035 (28.5%)	7391 (27.2%)
Overweight	21,975 (26.4%)	15,458 (27.5%)	6517 (24.0%)
Obese	34,041 (40.8%)	22,151 (39.4%)	11,890 (43.7%)
**Type 1 Diabetes**	1402 (1.7%)	910 (1.6%)	492 (1.8%)
**Type 2 Diabetes**	20,724 (24.9%)	14,414 (25.7%)	6310 (23.2%)
**Townsend quintile**			
1 Least deprived	17,908 (21.5%)	12,298 (21.9%)	5610 (20.6%)
2	17,675 (21.2%)	12,108 (21.6%)	5567 (20.5%)
3	18,207 (21.8%)	12,177 (21.7%)	6030 (22.2%)
4	16,973 (20.4%)	11,216 (20.0%)	5757 (21.2%)
5 Most Deprived	12,613 (15.1%)	8370 (14.9%)	4243 (15.6%)

a) Described as people who did not fit into any of the named categories.

b) Medications grouped: antiplatelet agents, statins, beta-blockers, ACE inhibitors.

c) Common mental illness includes depression, generalised anxiety disorder and panic disorder, obsessive-compulsive disorder and body dysmorphic disorder, post-traumatic stress disorder and social anxiety disorder.

d) Serious mental illness includes psychotic and non-psychotic disorders.

e) Categorised as index event occurring after March 2004.

#### Intervention subcategories

We analysed the frequency of each intervention recorded within the first year following the index event (Table 6). The most prevalent intervention documented was smoking cessation education, with over 64% of people (*N* = 53,469) receiving this code in their GP records. Post-QOF introduction, smoking cessation education remained predominant, with over 75.4% of people receiving it within one year of their stroke.

**Table 6:** Percentage of the population that received this form of intervention within one year of the index event

	Education	Pharma	Referral	Brief	Counselling	No Intervention
Whole Cohort (*n* = 83,376)	53,469 (64.1%)	8611 (10.3%)	2534 (3.0%)	402 (0.5%)	157 (0.2%)	27,207 (32.6%)
Abstinence within one year (*n* = 30,297)	23,266 (76.8%)	1608 (5.3%)	493 (1.6%)	87 (0.3%)	34 (0.1%)	6293 (20.8%)
Continued smoking within one year (*n* = 53,079)	30,203 (56.9%)	7003 (13.2%)	2041 (3.8%)	315 (0.6%)	123 (0.2%)	20,914 (39.4%)
CVD Death (*n* = 5,483)	2796 (51.4%)	364 (6.7%)	90 (1.7%)	15 (0.3%)	3 (0.1%)	2511 (45.8%)
Mental Illness						
No Mental Illness (*n* = 63,959)	40,687 (63.6%)	5856 (9.2%)	1845 (2.9%)	285 (0.4%)	116 (0.2%)	21,403 (33.5%)
Common Mental Illness (*n* = 17,525)	11,577 (66.1%)	2489 (14.2%)	623 (3.6%)	108 (0.6%)	37 (0.2%)	5196 (29.6%)
Serious Mental Illness (*n* = 1,892)	1205 (63.7%)	266 (14.1%)	66 (3.5%)	9 (0.5%)	4 (0.2%)	608 (32.1%)
Townsend quintile						
1 Least deprived (*n* = 17,908)	11,840 (66.1%)	1340 (7.5%)	348 (1.9%)	61 (0.3%)	31 (0.2%)	5610 (31.5%)
2 (*n* = 17,675)	11,627 (65.8%)	1621 (9.2%)	511 (2.9%)	73 (0.4%)	31 (0.2%)	5567 (31.5%)
3 (*n* = 18,207)	11,562 (63.5%)	1978 (10.9%)	546 (3.0%)	94 (0.5%)	42 (0.2%)	6030 (33.1%)
4 (*n* = 16,973)	10,593 (62.4%)	1988 (11.7%)	566 (3.3%)	87 (0.5%)	33 (0.2%)	5757 (33.9%)
5 Most Deprived (*n* = 12,613)	7847 (62.2%)	1684 (13.4%)	563 (4.5%)	87 (0.7%)	20 (0.2%)	4243 (33.6%)
After QOF (*n* = 65,078)	49,062 (75.4%)	7373 (11.3%)	2414 (3.7%)	402 (0.6%)	150 (0.2%)	13,870 (21.3%)
Before QOF (*n* = 18,291)	4407 (24.1%)	1238 (6.8%)	120 (0.7%)	0 (0.0%)	7 (< 1%)	13,337 (72.9%)

**Fig. 2:**
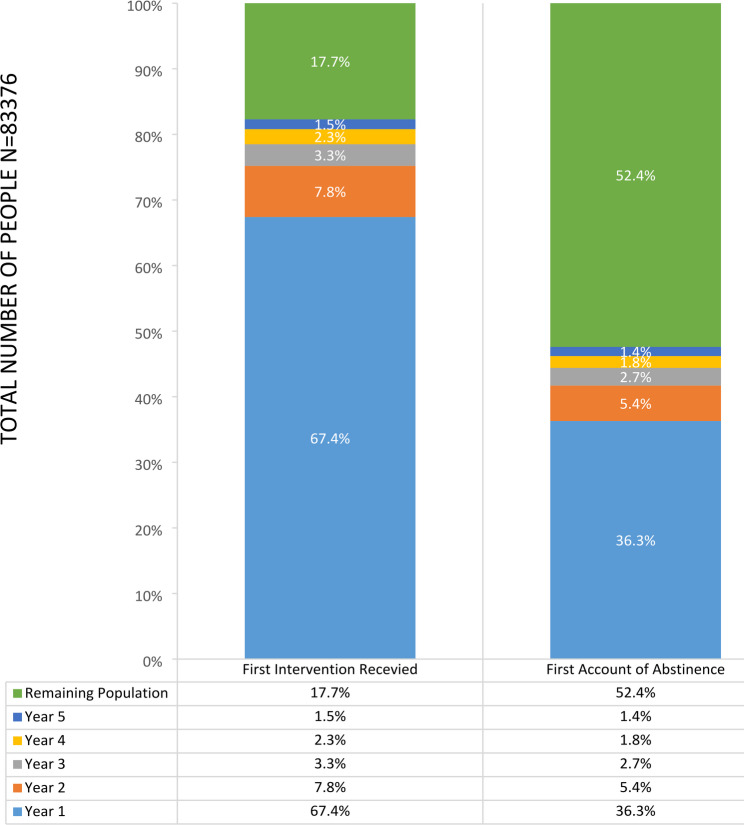
Bar chart illustrating the percentage who were first recorded as having received an intervention and the percentage who achieved abstinence categorised by year from index event of stroke

We examined the proportion of people receiving smoking cessation interventions within the initial five years following their stroke, and in which year (Fig. 2). Similar to people with CHD the rate of receiving a first intervention following the index event decreased annually. By the fifth year, 17.7% of the population had not received any smoking cessation intervention since their stroke. Beyond the fifth year, 52.4% of the population continued to smoke.

When analysing the population based on the presence of mental illness, 37.6% of people without mental illness achieved abstinence within the first year post-index event, compared to 33.1% with Common Mental Illness CMI and 23.4% with SMI (see Table 7).

**Table 7:** Baseline characteristics of people with stroke split by presence and categories of mental illness in database

Characteristic	No Mental Illness (*n* = 63,959)	Common Mental Illness (*n* = 17,525)	Serious Mental Illness (*n* = 1892)
**Age**			
Aged 18/30	73 (0.1%)	21 (0.1%)	5 (0.3%)
Aged 31/40	1005 (1.6%)	431 (2.5%)	57 (3.0%)
Aged 41/50	5416 (8.5%)	232.2 (13.2%)	252 (13.3%)
Aged 51/60	12,405 (19.4%)	4494 (25.6%)	513 (27.1%)
Aged 61/70	18,512 (28.9%)	4682 (26.7%)	572 (30.2%)
Aged 71/80	17,601 (27.5%)	3740 (21.3%)	386 (20.4%)
Aged 81/90	8095 (12.7%)	1674 (9.6%)	102 (5.4%)
Aged 91/110	852 (1.3%)	161 (0.9%)	5 (0.3%)
**Female**	25,520 (39.9%)	9437 (53.8%)	917 (48.5%)
**Ethnicity**			
White	42,988 (67.2%)	12,529 (71.5%)	1310 (69.2%)
Indian	605 (0.9%)	90 (0.5%)	20 (1.1%)
Pakistani	538 (0.8%)	102 (0.6%)	24 (1.3%)
Bangladeshi	374 (0.6%)	76 (0.4%)	17 (0.9%)
Other Asian	240 (0.4%)	54 (0.3%)	8 (0.4%)
Caribbean	699 (1.1%)	115 (0.7%)	47 (2.5%)
Black African	270 (0.4%)	45 (0.3%)	10 (0.5%)
Chinese	67 (0.1%)	12 (0.1%)	o (0.0%)
Other^a^	575 (0.9%)	123 (0.7%)	15 (0.8%)
Missing	17,603 (27.5%)	4379 (24.9%)	441 (23.3%)
**Abstinence within one year of index event**	24,055 (37.6%)	5800 (33.1%)	442 (23.4%)
**Current use of common secondary CVD Medication** ^b^	62,471 (97.7%)	17,139 (97.8%)	1813 (95.8%)
**Lung Cancer**	2366 (3.7%)	525 (3.0%)	56 (3.0%)
**Head and Neck Cancer**	235 (0.4%)	77 (0.4%)	5 (0.3%)
**COPD**	13,555 (21.2%)	4451 (25.4%)	455 (24.0%)
**Asthma**	7838 (12.3%)	3055 (17.4%)	313 (16.5%)
**Congestive Cardiac Failure**	7218 (11.3%)	1740 (9.9%)	152 (8.0%)
**Hypertension**	39,366 (61.5%)	10,086 (57.6%)	926 (48.9%)
**Terminal Illness**	234 (0.4%)	58 (0.3%)	10 (0.5%)
**Atrial Fibrillation**	11,900 (18.6%)	2613 (14.9%)	187 (9.9%)
**Index Event after Introduction of QOF** ^c^	48,867 (76.4%)	14,650 (83.6%)	1561 (82.5%)
**BMI Category**			
Underweight	3022 (4.7%)	832 (4.7%)	80 (4.2%)
Normal weight	18,490 (28.9%)	4365 (24.9%)	571 (30.2%)
Overweight	17,280 (27.0%)	4211 (24.0%)	484 (25.6%)
Obese	25,167 (39.3%)	8117 (46.3%)	757 (40.0%)
**Type 1 Diabetes**	976 (1.5%)	391 (2.2%)	35 (1.8%)
**Type 2 Diabetes**	15,658 (24.5%)	4555 (26.0%)	511 (27.0%)
**Townsend quintile**			
1 Least deprived	14,439 (22.6%)	3218 (18.4%)	251 (13.3%)
2	13,915 (21.8%)	3468 (19.8%)	292 (15.4%)
3	13,814 (21.6%)	3974 (22.7%)	419 (22.1%)
4	12,560 (19.6%)	3946 (22.5%)	467 (24.7%)
5 Most Deprived	9231 (14.4%)	2919 (16.7%)	463 (24.5%)

a) Described as people who did not fit into any of the named categories.

b) Medications grouped: antiplatelet agents, statins, beta-blockers, ACE inhibitors.

c) Categorised as index event occurring after March 2004.

### Impact of smoking cessation treatment on stopping smoking

#### CHD population

There was evidence that any intervention was associated with abstinence within one year of the index event OR 1.41 (95% CI 1.36, 1.45), full model in supplementary. Receiving education was associated with an increased likelihood while all other interventions were associated with a reduced risk of achieving abstinence.

People were less likely to stop smoking if they had either CMI or SMI, regardless of intervention status. People with mental illness were slightly more likely to have experienced a smoking cessation intervention, although imprecision for SMI meant confidence intervals encompassed one (Table 8). Subgroup analysis revealed that people showed greater likelihood of cessation with recorded interventions compared to no recorded intervention regardless of mental illness status. Sensitivity analysis comparing pre- versus post-QOF interventions, pre-QOF records were associated with significantly increased likelihoods of abstinence (OR 5.09, 95% CI [4.84, 5.35]).

**Table 8:** Association between recorded interventions and smoking cessation in people with CHD and people with stroke

Category	CHD Odds Ratio (OR)	CHD 95% CI	Stroke Odds Ratio (OR)	Stroke 95% CI
**Effects of Intervention Types on Likelihood of Abstinence**				
Education	1.31	[1.27, 1.34]	1.38	[1.33, 1.43]
Prescription	0.66	[0.61, 0.71]	0.49	[0.42, 0.56]
Referral	0.45	[0.42, 0.49]	0.42	[0.37, 0.47]
Brief advice	0.49	[0.40, 0.60]	0.50	[0.39, 0.65]
Counselling	0.65	[0.46, 0.92]	0.80	[0.53, 1.22]
**Any Intervention (any recorded vs. none)**				
**Intervention**	1.41	[1.36, 1.45]	1.49	[1.43, 1.55]
**Effects of Mental Illness on Likelihood of Abstinence (no mental illness as reference)**				
Common Mental Illness	0.78	[0.75, 0.80]	0.90	[0.86, 0.94]
Serious Mental Illness	0.50	[0.45, 0.56]	0.60	[0.53, 0.68]
**Effects of Mental Illness on Likelihood of Recorded Intervention (no mental illness as reference)**				
Common Mental Illness	1.08	[1.04, 1.13]	0.89	[0.86, 0.94]
Serious Mental Illness	1.07	[0.96, 1.20]	0.59	[0.53, 0.68]
**Effects of Recorded Intervention on Likelihood of Abstinence (no intervention as reference)**				
No Mental Illness	1.49	[1.44, 1.54]	1.62	[1.55, 1.71]
Common Mental Illness	1.07	[0.99, 1.15]	1.11	[1.02, 1.21]
Serious Mental Illness	1.31	[0.99, 1.71]	1.43	[1.05, 1.95]
**Effects of Interventions before QOF on Likelihood of abstinence (post QOF as reference)**				
Before QOF	5.09	[4.84, 5.35]	4.44	[4.07, 4.86]

#### Stroke population

Any intervention was associated with abstinence within one year after the index event OR 1.49 (95% CI 1.43, 1.55), full model in supplementary. Education was associated with a higher likelihood and all other interventions a lower likelihood of cessation.

People were less likely to stop smoking if they had either CMI or SMI, regardless of intervention status. There was also lower likelihood of recorded intervention among those with CMI and SMI compared to no mental illness (Table 8). Subgroup analysis revealed that people showed greater likelihood of cessation with recorded interventions compared to no recorded intervention across mental illness categories. Sensitivity analysis comparing pre- versus post-QOF interventions, pre-QOF records were associated with significantly increased likelihoods of smoking abstinence (OR 4.44, 95% CI [4.07, 4.86]).

## Discussion

### Main findings

This study investigated trends in smoking cessation interventions in primary care for people diagnosed with CHD and stroke, in the context of the QOF. People who had records of smoking cessation interventions in their GP records following their CHD or stroke event were more likely to quit smoking than people who did not have recorded smoking cessation interventions. Subgroup analyses revealed that people living with CVD and mental illness were less likely to cease smoking compared to those with CVD alone, regardless of whether they received a smoking cessation intervention or not. However, there was some evidence from the stroke population that although overall abstinence rates were not as high in people with a recorded mental illness their chances of quitting successfully were still improved by intervention (evidence from CHD patients was not clear; results should be treated with caution due to relatively small sample sizes). The introduction of the QOF was associated with an increase in recording of interventions for smoking cessation, but occurrence of such interventions predicted smoking abstinence much more strongly before rather than after the introduction of QOF. This raises questions about whether QOF improved clinical practice or simply incentivised coding behaviours, as seen in other areas of care.

### Interpretation and limitations

We contrasted active support to achieve cessation compared with advice or education alone. Our findings showed that pharmacological support was associated with a lower likelihood of smoking abstinence, whereas trial data show that such support increases smoking cessation [6, 36]. A network meta-analysis of RCTs conducted in people with CVD found that pharmacological support, alongside individual and telephone counselling, increased smoking cessation [37]. This discrepancy likely reflects real-world challenges, such as differences in patient adherence or selection biases, with GPs potentially targeting pharmacological support to people who had more severe smoking addiction who find it more difficult to quit smoking.

Furthermore, working with electronic health records (EHR) presents inherent limitations [38]. EHRs cannot accurately capture the nuances of GP practice and the motivations behind intervention decisions. The challenge of deciphering the meaning behind each code in GP databases further complicates the interpretation of results. While the study provides insights into coding frequencies, it does not elucidate the context behind when and why a GP will use one code over another, and what their actual behaviour was which led to the recording of that code. Coding behaviours are influenced by the QOF but may not accurately capture the delivery or quality of interventions. For example, education interventions may have been recorded for individuals who had already ceased smoking, whereby GPs provide education about not going back to smoking to such people. Furthermore, qualitative research could help elucidate why certain codes are used and uncover the motivations behind GP decisions, providing valuable context beyond the quantitative data.

While this study made the assumption that patients without SNOMED codes for mental illness were presumed to be without mental illness. This could have led to underreporting or miscoding of mental health conditions, particularly before the introduction of the QOF in 2004. However, the primary analysis in this study used data from 2004 onwards, when the QOF incentivised accurate recording of mental health status. Research using The UK Health Improvement Network (THIN) shows that SMI is generally well-recorded in GP databases, with a significant improvement post-QOF [39]. Nevertheless, some patients with mental health conditions may not have sought care for mental illness or may have received a delayed diagnosis, contributing to potential residual information bias.

The finding that approximately half of patients were still smoking five years after an initial intervention highlights a significant unmet need. Given that it may take over 30 quit attempts for some people to stop smoking [40], long-term support systems should be in place to facilitate quit attempts. Efforts could focus on incentivising types of language used when offering support as studies have found that certain GP communication strategies are more likely to lead to acceptance of smoking cessation interventions [41]. While we used a conservative criteria to define smoking cessation in this study, it is possible that the true number of people who successfully quit smoking has been underestimated. Knowing the limitations of EHRs regarding coding of smoking outcomes [42], this study prioritised the ability to best account for people who have truly stopped smoking. Qualitative and ethnographic research could better provide valuable insights into how to reach the remaining patients who are still smoking.

Given the benefits of smoking cessation both on mental health [19] and physical health [3], it is worrying to see that people living with both CVD and mental illness are stopping smoking at a lower rate than people without mental illness. Our study demonstrated that individuals living with both CVD and mental illness were less likely to cease smoking than those with CVD alone, regardless of the receipt of smoking cessation interventions. This finding likely underscores the complex interplay between physical and mental health in influencing smoking cessation outcomes.

A wider holistic approach might be necessary to understand how best to tailor health care for those living with multimorbidity. People with mental illness often face additional barriers, such as higher dependence on nicotine [43, 44] and increased life stressors [45] which likely make it harder to quit. Moreover, healthcare providers may hesitate to promote smoking cessation to this group due to misguided concerns about exacerbating mental health symptoms [26, 27] —though research has shown that these concerns are generally unfounded [19, 46]. This disparity suggests a critical need for cessation strategies that address the unique challenges faced by this population, ensuring interventions are both accessible and appropriately adapted to their needs. Given that evidence-based smoking cessation pharmacotherapy and non- pharmacotherapy smoking cessation interventions work for people living with CVD [37] and people living with mental health conditions [47, 48], perhaps the focus should be on understanding how to motivate GPs to give evidence-based smoking cessation interventions and more importantly implementing the interventions for patients with multiple comorbidities.

The increase in recorded advice but not evidence-based smoking cessation interventions and abstinence questions the effectiveness of the QOF programme, suggesting that it may have led to only improved coding rather than effective clinical action, which other studies have suggested [29, 49]. An interrupted time series analysis (ITSA) could be used to ascertain the true impact of QOF on population-level smoking cessation rates in people living with CVD. This has already been done in the general population and found no change in prescribing pharmacotherapy for cessation [29]. The exclusion of the COVID-19 period is a limitation, as the pandemic likely disrupted smoking cessation services and patient behaviour. Since our primary focus was on understanding trends in recording GP behaviours, analysing COVID-19 data would be more appropriately done through an ITSA and is recommended for future research.

### Generalisability

Currently, the QOF asks GPs to ‘offer support and treatment’, but the business rules allow GPs to ‘offer smoking cessation advice’ as equivalent. GP advice often on the harms of smoking or the benefits of quitting [50] less likely to increase smoking attempts compared to providing practical assistance [30]. Evidence suggests that brief, opportunistic interventions—such as the “very brief advice” (VBA) model—can significantly increase quit attempts when delivered in clinical settings [30, 51]. The VBA approach follows the 3 A’s:

1. **ASK**: Identify whether the patient smokes.
2. **ADVISE**: If the patient smokes, provide information on the most effective ways to quit (such as using medicinal aids, e-cigarettes, or behavioural support) and inform them that help is available.
3. **ACT**: Facilitate the patient’s access to evidence-based cessation services and quitting aids.

Prioritising and incentivising this approach may be more likely to increase patient engagement with smoking cessation services, translating into better outcomes. The current QOF incentive scheme may not be effectively improving patient care or smoking cessation rates, and a shift toward encouraging brief, evidence-based interventions could address this gap.

## Conclusions

This study highlights the gap between recorded smoking cessation interventions and actual quit rates, among people with CHD and stroke, particularly for those with mental illness. The QOF may have increased documentation of smoking cessation interventions but did not translate into higher smoking cessation rates. While interventions, particularly education, were well documented, cessation rates fell short of what RCT evidence would expect. Further research should explore why people with CVD and mental illness quit less and whether incentivising GPs for active interventions, rather than advice alone, would improve outcomes. Evidence suggests that combining pharmacological and behavioural interventions for smoking cessation maximises quitting success, yet the QOF currently allows GPs to fulfil requirements through advice alone. A shift toward incentivising evidence-based interventions, could improve long-term smoking abstinence rates. Policymakers and healthcare providers should focus on identifying how to better promote evidence-based strategies in primary care to ensure that those most at risk receive the support they need to quit smoking.

## Electronic supplementary material

Below is the link to the electronic supplementary material.

Supplementary Material 1

## Data Availability

To guarantee the confidentiality of personal and health information of patients, only the named authors have had full access to the data during the study, in accordance with the relevant licence agreements. Information on access to the QResearch data is available on the QResearch website (www.qresearch.org).
