# Supplementary material for: Investigating the association between recorded smoking cessation interventions and smoking cessation in people living with cardiovascular disease using UK general practice data
Source: BMC Prim Care. 2025 May 1;26:141. doi: 10.1186/s12875-025-02843-9 (PMC12044739; doi:10.1186/s12875-025-02843-9)
Supplement: Supplementary file 1 — Supplementary Material 1 [file 12875_2025_2843_MOESM1_ESM.docx]

Full model FOR CHD predicting abstinence

|  | Odds ratio | Std. err. | 95% CI Lower | 95% CI Upper |
| --- | --- | --- | --- | --- |
| Smoking Cessation Intervention Recorded | 1.41 | 0.02 | 1.36 | 1.45 |
| Prescription of Varenicline within 1 year | 0.28 | 0.01 | 0.25 | 0.31 |
| Prescription of Bupropion within 1 year | 0.19 | 0.03 | 0.14 | 0.27 |
| Prescription of NRT within 1 year | 0.45 | 0.01 | 0.43 | 0.47 |
| Hypertension | 1.05 | 0.01 | 1.03 | 1.08 |
| Atrial Fibrillation | 1.17 | 0.02 | 1.13 | 1.22 |
| Congestive Cardiac Failure | 1.11 | 0.02 | 1.07 | 1.15 |
| Asthma | 1.24 | 0.02 | 1.19 | 1.29 |
| COPD | 0.50 | 0.01 | 0.48 | 0.51 |
| Type 1 Diabetes | 1.06 | 0.06 | 0.96 | 1.19 |
| Type 2 Diabetes | 1.16 | 0.02 | 1.12 | 1.19 |
| Terminal Illness | 0.75 | 0.11 | 0.56 | 1.00 |
| Head and Neck Cancer | 0.71 | 0.09 | 0.55 | 0.92 |
| Lung Cancer | 0.64 | 0.03 | 0.59 | 0.70 |
| Psychiatric: ref no mental illness |  |  |  |  |
| Common Mental Illness | 0.78 | 0.01 | 0.75 | 0.80 |
| Serious Mental Illness | 0.50 | 0.03 | 0.45 | 0.56 |
| BMI: ref underweight |  |  |  |  |
| normal weight | 1.82 | 0.09 | 1.66 | 2.00 |
| overweight | 2.62 | 0.13 | 2.38 | 2.87 |
| obese | 2.51 | 0.12 | 2.29 | 2.75 |
| Ethnicity: ref White |  |  |  |  |
| Indian | 1.64 | 0.09 | 1.47 | 1.82 |
| Pakistani | 1.16 | 0.06 | 1.05 | 1.28 |
| Bangladeshi | 1.31 | 0.08 | 1.16 | 1.48 |
| Other Asian | 1.46 | 0.11 | 1.27 | 1.68 |
| Caribbean | 0.91 | 0.07 | 0.79 | 1.06 |
| Black African | 1.44 | 0.14 | 1.18 | 1.75 |
| Chinese | 0.97 | 0.19 | 0.67 | 1.42 |
| Other | 1.05 | 0.06 | 0.93 | 1.18 |
| Missing | 0.85 | 0.02 | 0.82 | 0.88 |
| Townsend Quintile Ref: Least deprived |  |  |  |  |
| 2 | 0.81 | 0.02 | 0.77 | 0.84 |
| 3 | 0.61 | 0.01 | 0.59 | 0.64 |
| 4 | 0.51 | 0.01 | 0.49 | 0.53 |
| 5 | 0.41 | 0.01 | 0.39 | 0.43 |
| Male | 1.04 | 0.02 | 1.01 | 1.07 |
| Cardiovascular Drugs | 2.99 | 0.20 | 2.63 | 3.40 |
| Age = ref 18/30 |  |  |  |  |
| Aged 31/40 | 4.79 | 3.62 | 1.09 | 21.08 |
| Aged 41/50 | 5.63 | 4.25 | 1.28 | 24.69 |
| Aged 51/60 | 7.42 | 5.60 | 1.69 | 32.53 |
| Aged 61/70 | 11.22 | 8.46 | 2.56 | 49.20 |
| Aged 71/80 | 15.90 | 11.99 | 3.63 | 69.71 |
| Aged 81/90 | 20.42 | 15.41 | 4.65 | 89.59 |
| Aged 91/110 | 25.94 | 19.70 | 5.85 | 114.95 |

Full model FOR Stroke

|  | Odds ratio | Std. err. | 95% CI Lower | 95% CI Upper |
| --- | --- | --- | --- | --- |
| Smoking Cessation Intervention Recorded | 1.49 | 0.03 | 1.43 | 1.55 |
| Psychiatric: ref no mental illness |  |  |  |  |
| Common Mental Illness | 0.90 | 0.02 | 0.86 | 0.94 |
| Serious Mental Illness | 0.60 | 0.04 | 0.53 | 0.68 |
| Prescription of Varenicline within 1 year | 0.35 | 0.03 | 0.31 | 0.41 |
| Prescription of Bupropion within 1 year | 0.23 | 0.07 | 0.13 | 0.41 |
| Prescription of NRT within 1 year | 0.41 | 0.01 | 0.39 | 0.44 |
| Hypertension | 1.13 | 0.02 | 1.09 | 1.17 |
| Atrial Fibrillation | 1.37 | 0.03 | 1.30 | 1.43 |
| Congestive Cardiac Failure | 1.10 | 0.03 | 1.04 | 1.17 |
| Asthma | 1.39 | 0.04 | 1.32 | 1.46 |
| COPD | 0.49 | 0.01 | 0.47 | 0.51 |
| Type 1 Diabetes | 0.89 | 0.06 | 0.77 | 1.03 |
| Type 2 Diabetes | 1.20 | 0.03 | 1.16 | 1.25 |
| Terminal Illness | 0.70 | 0.11 | 0.52 | 0.96 |
| Head and Neck Cancer | 0.88 | 0.13 | 0.66 | 1.17 |
| Lung Cancer | 0.65 | 0.03 | 0.58 | 0.72 |
| BMI: ref underweight |  |  |  |  |
| normal weight | 1.73 | 0.08 | 1.57 | 1.91 |
| overweight | 2.56 | 0.13 | 2.33 | 2.82 |
| obese | 2.14 | 0.10 | 1.95 | 2.35 |
| Ethnicity: ref White |  |  |  |  |
| Indian | 1.77 | 0.16 | 1.49 | 2.10 |
| Pakistani | 1.11 | 0.10 | 0.93 | 1.32 |
| Bangladeshi | 1.54 | 0.17 | 1.23 | 1.92 |
| Other Asian | 1.85 | 0.24 | 1.43 | 2.38 |
| Caribbean | 1.25 | 0.10 | 1.06 | 1.47 |
| Black African | 3.32 | 0.41 | 2.60 | 4.24 |
| Chinese | 1.88 | 0.48 | 1.13 | 3.10 |
| Other | 0.85 | 0.02 | 0.81 | 0.89 |
| Missing | 1.88 | 0.48 | 1.13 | 3.10 |
| Townsend Quintile Ref: Least deprived |  |  |  |  |
| 2 | 0.81 | 0.02 | 0.77 | 0.85 |
| 3 | 0.63 | 0.02 | 0.60 | 0.67 |
| 4 | 0.53 | 0.01 | 0.51 | 0.56 |
| 5 | 0.44 | 0.01 | 0.41 | 0.47 |
| Male | 0.95 | 0.02 | 0.92 | 0.99 |
| Cardiovascular Drugs | 1.92 | 0.13 | 1.68 | 2.19 |
| Age = ref 18/30 |  |  |  |  |
| Aged 31/40 | 0.50 | 0.24 | 0.20 | 1.27 |
| Aged 41/50 | 0.60 | 0.28 | 0.24 | 1.49 |
| Aged 51/60 | 0.74 | 0.35 | 0.30 | 1.85 |
| Aged 61/70 | 1.14 | 0.53 | 0.46 | 2.85 |
| Aged 71/80 | 1.79 | 0.83 | 0.72 | 4.45 |
| Aged 81/90 | 2.58 | 1.20 | 1.04 | 6.44 |
